# *KMT2C* promoter methylation in plasma‐circulating tumor DNA is a prognostic biomarker in non‐small cell lung cancer

**DOI:** 10.1002/1878-0261.12848

**Published:** 2020-12-25

**Authors:** Sofia Mastoraki, Ioanna Balgkouranidou, Emily Tsaroucha, Apostolos Klinakis, Vassilis Georgoulias, Evi Lianidou

**Affiliations:** ^1^ Analysis of Circulating Tumor Cells Lab of Analytical Chemistry Department of Chemistry University of Athens Greece; ^2^ 8th Department of Pulmonary Diseases ‘Sotiria’ General Hospital for Chest Diseases Athens Greece; ^3^ Biomedical Research Foundation Academy of Athens Greece; ^4^ Department of Medical Oncology IASO General Hospital Athens Greece; ^5^ Present address: Department of Experimental Radiation Oncology UT MD Anderson Cancer Center Houston TX USA

**Keywords:** cfDNA, epigenetic biomarkers, *KMT2C*, liquid biopsy, *MLL3*, NSCLC

## Abstract

MLL3 histone methyltransferase, encoded by the *KMT2C* gene, is a tumor suppressor that has an essential role in cell‐type‐specific gene expression. We evaluated the prognostic significance of *KMT2C* promoter methylation as a circulating epigenetic biomarker in plasma cell‐free DNA (cfDNA) in non‐small cell lung cancer (NSCLC). We examined the methylation status of *KMT2C* promoter using a novel highly specific and sensitive real‐time methylation‐specific PCR (MSP) assay in (a) operable NSCLC: 48 fresh‐frozen NSCLC tissues, their corresponding adjacent non‐neoplastic tissues, and 48 matched plasma samples; (b) metastatic NSCLC: 91 plasma samples; and (c) 60 plasma samples from healthy donors (HD). *KMT2C* promoter methylation in plasma cfDNA was detected in 7/48 (14.6%) patients with operable and in 18/91 (19.8%) patients with advanced NSCLC but in none (0/60, 0%) of the plasma samples from HD. In operable NSCLC, in corresponding adjacent non‐neoplastic tissue samples, *KMT2C* promoter methylation was detected in 3/48 (6.3%) cases. Moreover, in operable NSCLC, *KMT2C* promoter methylation in plasma cfDNA was related to reduced disease‐free survival (ΗR = 0.239; *P* = 0.001) and worse overall survival (OS; HR = 0.342, *P* = 0.023). In metastatic NSCLC, *KMT2C* promoter methylation in plasma cfDNA was related to worse progression‐free survival (PFS; HR = 0.431; *P* = 0.005) and worse OS (HR = 0.306; *P* < 0.001). Our data strongly suggest that the detection of *KMT2C* promoter methylation in plasma cfDNA predicts poor prognosis in patients with both operable and metastatic NSCLCs. *KMT2C* promoter methylation in plasma cfDNA therefore merits further evaluation and validation as a noninvasive circulating epigenetic biomarker.

## Graphical abstract

Liquid biopsies are emerging noninvasive diagnostic tools in cancer. MLL3 histone methyltransferase is a transcriptional regulator encoded by the tumor suppressor *KMT2C* gene. We evaluated the prognostic significance of *KMT2C* epigenetic changes in plasma of NSCLC patients. The detection of *KMT2C* promoter methylation in cfDNA predicts poor outcomes in NSCLC and merits further evaluation as a circulating epigenetic biomarker.

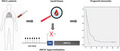

**Abbreviations**
cfDNA: cell‐free DNA
CTCs: circulating tumor cells
gDNA: genomic DNA
HD: healthy donors
MSP: methylation‐specific PCR
NSCLC: non‐small cell lung cancer
SB: sodium bisulfite

## 1 Introduction

Lung cancer remains one of the most common cancers and lethal diseases in the world [1]. Non‐small cell lung carcinoma (NSCLC), the major subtype of lung cancer, can be subdivided into three subtypes: lung adenocarcinoma (ADC), squamous cell carcinoma (SCC), and large‐cell carcinoma [2]. The detection of NSCLC at an early stage can favor prognosis; however, there is still a lack of good prognostic biomarkers that could predict clinical outcome. A significant number of actionable genetic driver mutations and translocations have been revealed for targeted therapy for NSCLC patients [3, 4, 5].

Nowadays, noninvasive or minimally invasive diagnostic tools based on liquid biopsy have significantly contributed to the successful management of lung cancer patients [6]. Liquid biopsy provides a noninvasive way for real‐time monitoring of tumor evolution, and therapeutic efficacy, as well as an important insight into tumor heterogeneity [7]. In addition, liquid biopsy has recently revealed important biological mechanisms driving metastatic initiator cells in lung cancer [8, 9, 10]. Cell‐free DNA (cfDNA) analysis is already being applied in routine clinical practice for the detection of EGFR mutations in plasma samples of NSCLC patients to stratify those patients who may be benefited from targeted therapies [11, 12].

Beyond gene mutations, recent advances in lung cancer epigenetic research effectively provide a promising step toward the discovery of novel epigenetic biomarkers in NSCLC, since DNA methylation alterations are frequently observed and have diverse implications in carcinogenesis, diagnosis, and prediction [13, 14, 15]. DNA methylation is an ideal source of candidate biomarkers due to its stability, its virtually universal presence, and amenability to measurement [13, 16]. Methylation of cancer‐related genes in lung cancer patients has already been detected in various biological samples, including bronchoscopic washings/brushings, sputum samples, plasma, and serum [13]. Analysis of DNA methylation markers in biological fluids of lung cancer patients could lead to the discovery of putative epigenetic biomarkers for diagnosis, prognosis, risk assessment, and disease monitoring [17, 18]. We were the first to report epigenetic changes in circulating tumor cells (CTCs) [17, 19, 20] and to demonstrate a close correlation between DNA methylation in CTCs and paired plasma cfDNA [17, 21]. We have also shown the prognostic value of *BRMS1* and *SOX17* in plasma cfDNA from NSCLC patients [22, 23].

The KMT2 family [also known as mixed‐lineage leukemia (MLL)] has important functions in transcriptional regulation; KMT2 enzymes bind to several promoters, enhancers, and other regulatory regions [24]. Specifically, the functionally redundant H3K4me1/2 methyltransferases MLL3 (KMT2C) and MLL4 (KMT2D) are enriched on both silent and active enhancers [25]. A recent study indicated that both these enzymes facilitate enhancer RNA synthesis and transcription from promoters independently of H3K4 monomethylation [26]. In normal cells, both MLL3 and MLL4 are essential for enhancer activation and cell‐type‐specific gene expression during cell differentiation [25, 27]. MLL3/MLL4 is contributed to the formation of super‐enhancers (SEs) by binding CBP/p300 and that enhancer priming sequentially shapes the dynamic enhancer landscapes during adipogenesis [28].

A past study from Hu *et al*. in human HCT116 cells showed that 70% of putative enhancers present significant loss in H3K4me1 levels in MLL3^Δset^/4^Δset^ cells. Gene ontology (GO) analysis of genes proximal to the MLL3/4‐specific enhancers revealed that many of these genes are implicated in intracellular signaling, whereas MLL3/4‐independent enhancers revealed include genes that are implicated in intracellular signaling [29]. In a more recent study from Rampias *et al*., KMT2C was found to bind to enhancers proximally located to 2808 genes, many of which were involved in cell adherence to the epithelial basement membrane (ITGB1, ITGB6, RHOB, MMP7); extracellular matrix organization (LOXL2, LOXL4, TIMP4); and epithelial development and differentiation (SMAD6, SOX2, EREG, WNT11, BMP2) [30]. KMT2C silencing led to significant H3K27ac loss in these enhancers and increased binding by the transcription factors JUNB, TEAD, RUNX1, and MAFA [30].

High *KMT2C* mutational burden in several epithelial cancers implies a role as a tumor suppressor. Exome sequencing of human tumors has revealed an increased number of disease‐specific mutations in the KMT2/MLL gene family that places them among the most frequently mutated genes in human cancer[24]. Up to 60% of known *KMT2C* mutations have been specifically identified in carcinomas of the lungs, large intestine, breast [31], uterus, and urinary tract [32]. Recently, it has been shown that *KMT2C* genetic polymorphisms were also associated with larynx cancer in a Chinese population [33]. Many studies have also suggested that *KMT2C* expression could be used as a prognostic biomarker in gastric [34], pancreatic [35], and ER^+^ breast cancer [36]. Finally, a role of *KMT2C* as a 7q haplo‐insufficient tumor suppressor gene has been proposed in acute myeloid leukemia in mice, by impairing the differentiation of HSPC and cooperating with other established AML lesions [37].

A meta‐analysis of RNA‐seq data from The Cancer Genome Atlas (TCGA) Consortium has shown that *KMT2C* expression is downregulated in NSCLC in comparison with normal tissue, and reduced *KMT2C* expression in patients with NSCLC was correlated with DNA hypermethylation within a CpG island (chr7:152435133–152437025, assembly GRCh38/hg38, ENCODE) of the *KMT2C* proximal promoter [30].

In this study, we evaluated for the first time the prognostic significance of KMT2C promoter methylation in NSCLC. We analyzed *KMT2C* promoter methylation in DNA samples from fresh‐frozen NSCLC primary tissues, matched adjacent noncancerous tissues and plasma cfDNA from operable NSCLC patients, plasma cfDNA from metastatic NSCLC patients, and healthy individuals. Our data strongly suggest that the detection of *KMT2C* promoter methylation in plasma cfDNA predicts poor prognosis in patients with both operable and metastatic NSCLCs.

## 2 Materials and methods

**Fig 1:**
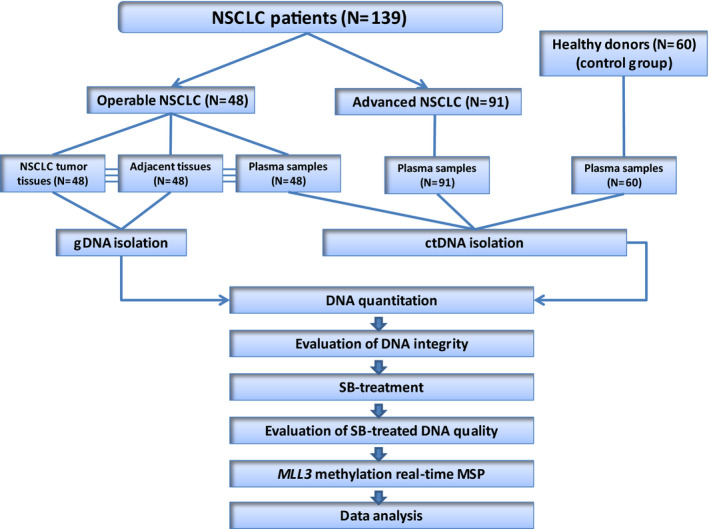
Experimental flowchart of the study.

The workflow of the study is shown in Fig. 1.

### 2.1 Patients

Blood samples were collected from two different cohorts of patients with histologically confirmed NSCLC: (a) a group consisting of 48 patients with operable (early stage: IA–IIIA) and (b) a group consisting of 91 patients with metastatic NSCLC (stage IV). Moreover, a cohort of 60 age‐matched male and female healthy individuals was used as control group. In this group, 48 fresh‐frozen tissues, corresponding adjacent non‐neoplastic tissues, and matched plasma samples (collected before surgery) were analyzed. In the metastatic NSCLC group, plasma was collected before the initiation of first‐line chemotherapy. All tissue specimens and plasma samples were collected randomly and not on purpose to balance the smoking status. The study was conducted in accordance with the 1964 Declaration of Helsinki and was approved by the ethics and scientific committee of ‘Sotiria’ General Hospital for Chest Diseases, Athens. All participating patients signed an informed consent form to participate in the study. The collection of samples was performed between 2004 and 2006. In the operable NSCLC group, samples were collected from 37 men and 11 women (median age: 61 years), all diagnosed with operable (stages I–III) NSCLC; 28 patients were diagnosed with ADC, 19 with SCC, and one with undifferentiated NSCLC; in this group, the majority of patients (60.4%) were smokers and suffered from mild‐to‐moderate chronic obstructive pulmonary disease according to pulmonary function tests that were included as part of the standard preoperative evaluation of the patients. Moreover, all patients received adjuvant platinum‐based chemotherapy according to the national guidelines. The main patients' characteristics are summarized in Table 1.

**Table 1:** Correlation of *KMT2C* promoter methylation status in plasma‐derived cfDNA samples with clinicopathological characteristics in patients with operable NSCLC. *P*‐values < 0.005 are considered statistically significant

Clinicopathological characteristic	Patients^a^	*KMT2C* promoter methylation (cfDNA)		*P*‐value^b^
		Positive (%)	Negative (%)	
Gender	*N* = 40			
Male	34	6/40 (15)	28/40 (70)	0.565
Female	6	0/40 (0)	6/40 (15)	
Age (median = 61, range: 47–78)	*N* = 34			
< 61	16	2/16 (12.5)	14/16 (87.5)	0.660
≥ 61	18	4/18 (22.2)	14/18 (77.8)	
Histology	*N* = 48			
ADC	28	4 (14.3)	24 (85.7)	0.907
SCC	19	3 (15.8)	16 (84.2)	
Undifferentiated	1	0 (0)	1 (100)	
Tumor size	*N* = 39			
< 5	18	0/18 (0)	18/18 (100)	0.235
≥ 5	21	3/21 (14.3)	18/21 (85.7)	
Stage	*N* = 41			
I, II	28	0/28 (0)	28/28 (100)	< 0.001
III, IV	13	7/13 (53.8)	6/13 (46.2)	
Lymph nodes	*N* = 41			
Negative	19	2/19 (10.5)	17/19 (89.5)	0.588
Positive	22	1/22 (4.5)	21/22 (95.5)	
Smoking status	*N* = 48			
Nonsmoker	19	1/19 (5.3)	18/19 (94.7)	0.034
Smoker	29	6/29 (20.7)	23/29 (79.3)	

^a^ In cases where the total number of patients is different, this is due to nonavailable clinical information.

^b^ Fisher's exact test.

### 2.2 Sample preparation

To avoid PCR contamination, different rooms, dedicated labware, and dedicated areas were used for all procedures. All DNA preparation and handling steps took place in specific laminar‐flow hoods under DNase‐free conditions.

#### 2.2.1 gDNA isolation from fresh‐frozen tissues

Tissue sections containing > 80% of tumor cells were used for DNA extraction and real‐time methylation‐specific PCR (real‐time MSP) analysis. Genomic DNA (gDNA) from NSCLC tissues and corresponding adjacent tissues was isolated using the DNeasy Blood and Tissue Kit (Qiagen, Hilden, Germany) according to the manufacturer's instructions. DNA concentration in all cases was measured with a NanoDrop 1000 Spectrophotometer (Thermo Scientific, USA) calibrated with a standard solution; isolated gDNA and sodium bisulfite (SB)‐treated DNA samples were stored at −80 °C until further use [17].

#### 2.2.2 cfDNA isolation from plasma

Whole blood samples were collected into venous blood collection tubes using EDTA as anticoagulant. Plasma was isolated within 2–4 h from sample collection by centrifugation at 530 ***g*** for 10 min at room temperature. Once isolated, plasma samples were centrifuged again at 2000 ***g*** for 10 min, before transferring into clean 2‐mL tubes, and were stored at −70 °C until time of processing. The High Pure Viral Nucleic Acid Kit (Roche Diagnostics, Mannheim, Germany) was used to extract cfDNA from 200 μL of plasma, according to the manufacturer's instructions [17, 19, 21, 22, 23].

### 2.3 Sodium bisulfite treatment

Genomic DNA samples were treated with SB, to convert all nonmethylated cytosines to uracil, while methylated cytosines were not converted, using the EZ DNA Methylation Gold Kit (Zymo Research, Irvine, CA, USA). Converted DNA was stored at –70 °C until use. In each SB reaction, dH_2_O and 100% methylated DNA were included as negative and positive controls, respectively [17, 19, 21, 22, 23].

### 2.4 Development and analytical validation of the real‐time MSP assay for *KMT2C* promoter methylation

#### 2.4.1 *In silico* primer design

We designed *in silico* primers in the *KMT2C* promoter region, specific for *KMT2C*‐MSP, using primer premier 5.00 software (Premier Biosoft, San Franscisco, CA, USA) avoiding the formation of stable hairpin structures, primer dimers, cross dimers, and false priming sites. The *in silico* validation was carried out using BLAST tool, in order to check their specificity and eliminate the risk of amplifying undesired sequences. For maximal discrimination between methylated and nonmethylated alleles, both primers contained several CpGs. Additionally, both primers contained T bases derived from modified nonmethylated C regions to allow discrimination and amplification of the converted from the unconverted DNA. Primers were synthesized by Integrated DNA Technologies (IDT, Leuven, Belgium, USA). Primer sequences are available upon request [17].

#### 2.4.2 Optimization of experimental conditions

The experimental conditions of real‐time MSP for *KMT2C* promoter methylation were first optimized in detail for the annealing temperature and time, then for the optimum concentrations of the primer pair, and finally for buffer, MgCl_2_, dNTP, and BSA concentrations (data not shown). Each MSP reaction was performed in the 96‐well plate LightCycler^®^ 480 System (IVD; Roche Molecular Diagnostics) in a total volume of 10 μL. One microliter of SB‐converted DNA was added to 9 μL reaction mixture containing 0.05 U·μL^−1^ GoTaq® Hot Start Polymerase (Promega, Maddison, WI, USA), 0.2× of the supplied PCR buffer, 2 mm of MgCl_2_, 0.2 mm of each dNTP (Thermo Fisher Scientific), 0.3 μg·μL^−1^ BSA, 0.3 μμ of the forward and reverse primers, and 1× LCGreen Plus Dye (Idaho Technology, Salt Lake City, Utah, USA). Finally, deionized water was added to a final volume of 10 μL. Real‐Time MSP protocol began with one cycle at 95 °C for 2 min followed by 45 cycles of 95 °C for 10 s, 63 °C for 20 s, and 72 °C for 20 s. Immediately after amplification, a rapid cooling cycle to 40 °C for 30 s was introduced in order to prepare the melting curve acquisition step. Real‐time fluorescence acquisition was set at the elongation step (72 °C). The following melting curve analysis included the steps of 55 °C for 10 s, 92 °C for 0 s with a ramp rate 0.11 °C·s^−1^ (acquisition mode: continuous), 92 °C for 1 min, and 40 °C for 1 min [17, 19, 21, 22, 23].

#### 2.4.3 Analytical specificity

To evaluate the analytical specificity of the *KMT2C* real‐time MSP, we initially tested the primers *in silico* and then in PCR, using gDNA (unconverted DNA) and SB‐modified human placental gDNA samples that were not methylated; no amplification of the *KMT2C* was observed. In contrast, amplification was observed only when SB‐treated DNA from the 100% methylated standard was used. The developed assay is highly specific since it can detect only SB‐treated *KMT2C*‐methylated sequences [17, 19, 21, 22, 23].

#### 2.4.4 Analytical sensitivity

**Fig 2:**
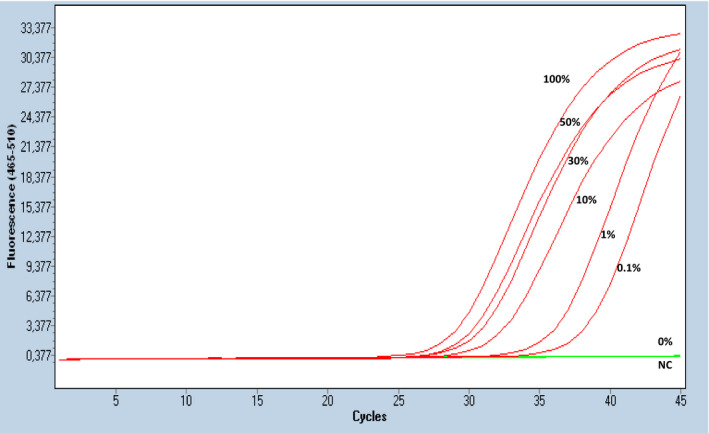
Analytical sensitivity of the developed real‐time MSP assay for *KMT2C* promoter methylation. All MSP done in triplicate.

The analytical sensitivity of the developed real‐time MSP was evaluated by using synthetic mixtures based on serial dilutions of SB‐converted DNA control samples (0% and 100% methylated) at various percentages of methylation (0.1%, 1%, 10%, 30%, and 50%). Each dilution was analyzed in triplicate, and our results indicate that the developed real‐time MSP assay for *KMT2C* could specifically and reliably detect the presence of 0.1% methylated *KMT2C* sequences, in the presence of 99.9% nonmethylated *KMT2C* sequences (Fig. 2) [17, 19, 21, 22, 23].

#### 2.4.5 Diagnostic specificity

The diagnostic specificity of the developed assay was evaluated by using sixty plasma samples from healthy donors (HDs). cfDNA was isolated from all these plasma samples, and the *KMT2C* methylation status was assessed by the developed specific and sensitive real‐time MSP assay; none of the HD plasma samples were methylated at the *KMT2C* promoter (0/60, 0%).

### 2.5 Quality control

We have recently evaluated the effect of pre‐analytical conditions on DNA methylation analyses in liquid biopsies, such as the time interval to plasma isolation, SB‐converted DNA storage conditions, and the validity of whole‐genome amplification for SB‐converted DNA [16]. Quality control checks were performed in all steps prior to sample analysis. In each step of the analytical procedure, we included appropriate positive and negative controls in order to ensure the quality and reproducibility of results. Before proceeding to the SB treatment and real‐time MSP, we assessed the integrity of gDNA in all samples by amplifying exon 20 of *PIK3CA* [38]. Only samples that were positive for *PIK3CA* exon 20 amplification were further processed to SB treatment. The quality of SB‐treated DNA was checked by a real‐time MSP assay for *β‐actin* (*ACTB*). To verify that we could specifically detect only *KMT2C*‐methylated sequences, we used three different controls: gDNA not subjected to SB conversion (unconverted DNA), human placental gDNA (Sigma‐Aldrich, Darmstadt, Germany, USA) submitted to SB conversion (placental converted DNA, 0% methylated) that was used as a negative real‐time MSP control after SB treatment, and the Universal Methylated Human DNA Standard (ZYMO Research, Irvine, CA, USA) that was used as fully methylated (100%) positive control [17, 19, 21, 22, 23].

### 2.6 Statistical analysis

Correlations between *KMT2C* methylation status and the clinicopathological characteristics of the patients were assessed using the chi‐square test of independence for data analysis. Disease‐free survival (DFS), progression‐free survival (PFS), and overall survival (OS) curves were calculated by using the Kaplan–Meier method, and comparisons were performed using the log‐rank test. Finally, univariate Cox regression analysis was performed to identify the risk of progression and death in *KMT2C* methylation‐positive patients. *P*‐values < 0.05 were considered statistically significant. Statistical analysis was performed by using the spss Statistics, version 25.0 (SPSS Inc., Chicago, IL, USA) [17, 19, 21, 22, 23].

## 3 Results

### 3.1 Operable NSCLC

**Fig 3:**
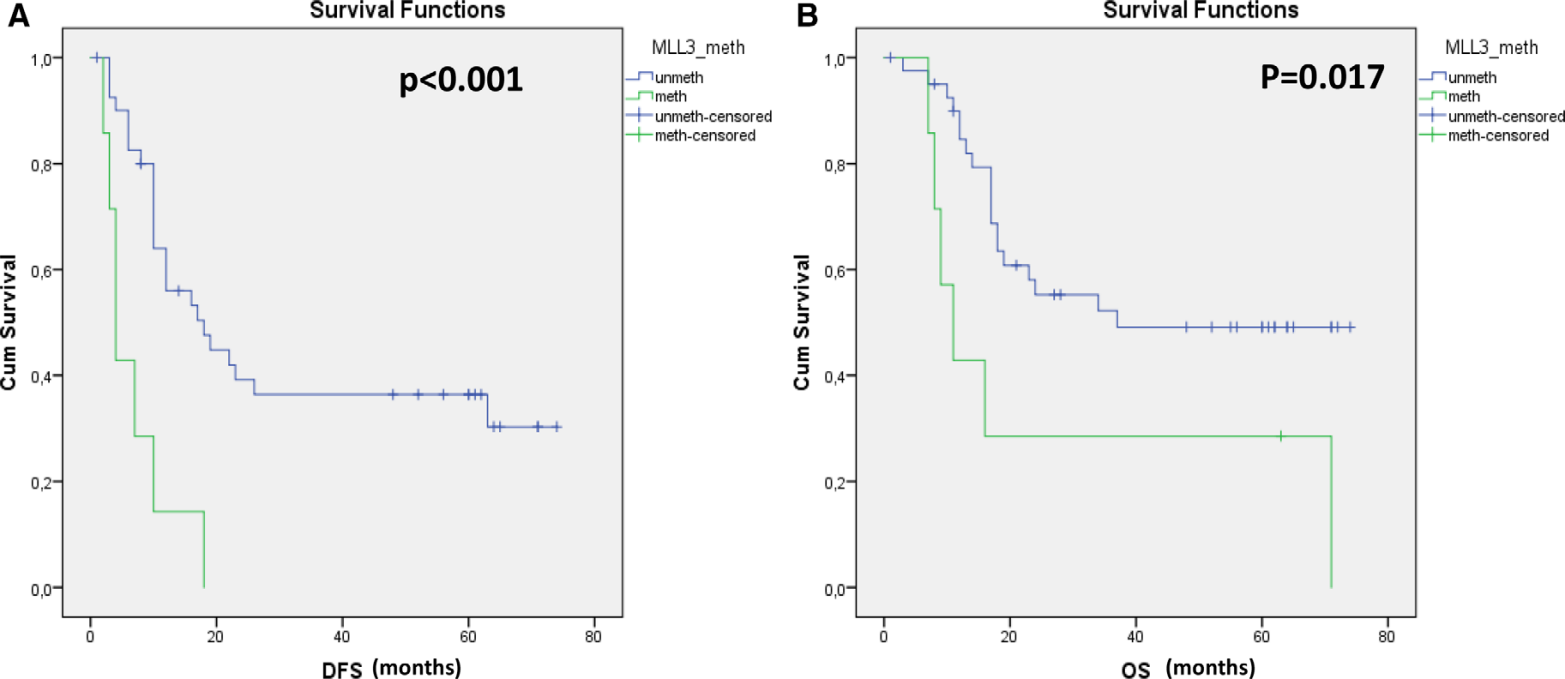
Kaplan–Meier estimates of operable NSCLC patients in relation to *KMT2C* promoter methylation in plasma‐cfDNA samples; (A) *KMT2C* methylation in relation to DFS (months) and (B) *KMT2C* methylation in relation to OS (months).

The methylation status of *KMT2C* promoter was first evaluated in 48 NSCLC fresh‐frozen primary tumors and their adjacent non‐neoplastic tissues. *KMT2C* was found methylated in 3/48 (6.3%) fresh‐frozen tissues and in 3/48 (6.3%) of the corresponding adjacent noncancerous tissues. In 2/3 cases, *KMT2C* was methylated in both primary tumor and corresponding adjacent noncancerous tissue, while one sample was positive only in the primary tumor but not in the corresponding adjacent tissue, while another sample was positive for *KMT2C* methylation only in the adjacent tissue but not in the paired primary tumor. We further evaluated *KMT2C* methylation status in matched plasma cfDNA samples; *KMT2C* methylation was observed in 7/48 (14.6%) plasma samples. Fisher's exact test revealed a statistically significant correlation between *KMT2C* promoter methylation and NSCLC stage as well as the smoking status of patients (*P* < 0.001 and *P* = 0.034, respectively; Table 1). After a median follow‐up period of 45 months (range: 1–73 months), 32/48 (66.7%) patients relapsed and 30/48 (62.5%) died due to disease progression. *KMT2C* promoter methylation was detected in 7/32 (21.9%) of patients that relapsed and in 6/30 (20%) of patients that died. The Kaplan–Meier estimates of the cumulative DFS and OS for NSCLC patients with methylated and nonmethylated *KMT2C* promoter methylation in plasma cfDNA were significantly different in favor of MSP‐negative patients (*P* < 0.001 and *P* = 0.017, log‐rank test, respectively; Fig. 3A,B). Univariate Cox regression analysis also revealed a significantly higher risk of progression (HR: 0.239, 95% CI: 0.099–0.575, *P* = 0.001) and death (HR: 0.342, 95% CI: 0.135–0.865, *P* = 0.023) in the *KMT2C* methylation‐positive compared with *KMT2C* methylation‐negative cfDNA samples from operable NSCLC patients.

To rule out the effect of advanced stage in predicting worse outcomes independently of *KMT2C* promoter methylation, we separated the unmethylated operable NSCLC group into early (*N* = 28, stages I–II) and advanced (*N* = 6, stages III–IV) patients and performed additional survival analysis. Although the end numbers of patients are low, our analysis revealed that stage on itself is insufficient to predict survival in the operable NSCLC cohort for both OS and DFS (*P* = 0.560 and *P* = 0.519, respectively; Table 1, Fig. S1). Lastly, multivariate Cox regression analysis including *KMT2C* methylation, LN status, smoking status, age, stage, tumor size, and gender in the operable NSCLC group showed that stage is not associated with a higher risk of progression or death, while *KMT2C* promoter methylation can potentially predict higher risk of death (results not shown). These findings further support our hypothesis that *KMT2C* promoter methylation in plasma can be used as a circulating prognostic biomarker, predictive of worse outcomes in NSCLC.

### 3.2 Metastatic NSCLC

**Fig 4:**
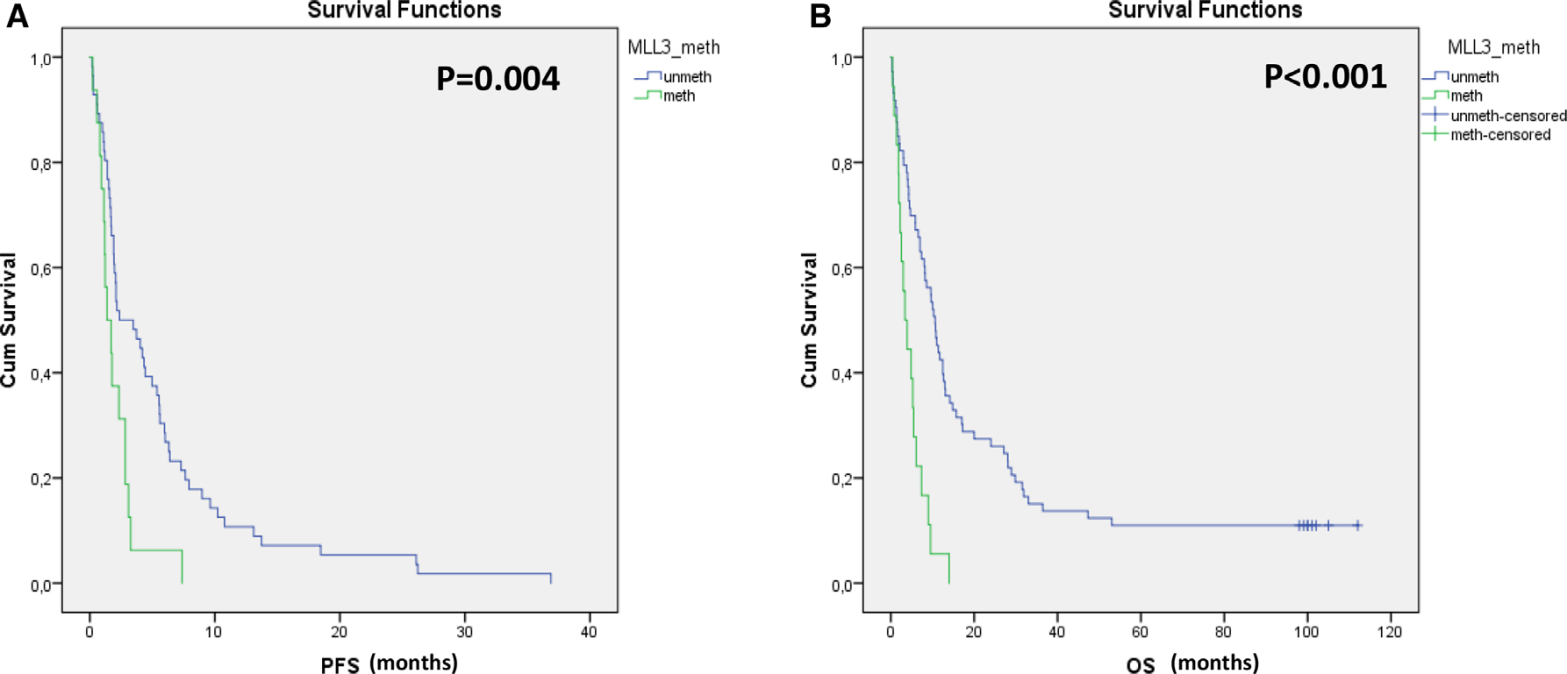
Kaplan–Meier estimates of advanced NSCLC patients in relation to *KMT2C* promoter methylation in plasma‐cfDNA samples; (A) *KMT2C* methylation in relation to PFS (months) and (B) *KMT2C* methylation in relation to OS (months).

Τhe prognostic significance of *KMT2C* promoter methylation in plasma cfDNA was subsequently evaluated in an independent cohort of 91 patients with metastatic NSCLC after a long‐term follow‐up. In this group, we found that *KMT2C* promoter was methylated in plasma‐derived cfDNA in 18/91(19.8%) cases. More specifically, after a median follow‐up of 52 months (range: 1–112 months), 87/91 (95.6%) patients relapsed and 83/91 (91.2%) patients died due to disease progression. All plasma samples that were found positive for *KMT2C* promoter methylation belonged to the group of patients who relapsed and died. Kaplan–Meier estimates of the cumulative PFS for patients with methylated and nonmethylated *KMT2C* promoter demonstrated a statistical significance (*P* = 0.004, log‐rank test; Fig. 4A). In parallel, the Kaplan–Meier estimates of the cumulative OS were also significantly different between the two groups (*P* < 0.001, log‐rank test; Fig. 4B). In addition, the univariate Cox regression analysis revealed that both PFS and OS were significantly different in patients with *KMT2C* promoter methylation (HR: 0.431, 95% CI: 0.239–0.779, *P* = 0.005, and HR: 0.306, 95% CI: 0.173–0.541, *P* < 0.001, respectively).

## 4 Discussion

Circulating tumor DNA provides a real‐time monitoring of tumor progression and is a unique and minimally invasive way to monitor response of solid tumors to anticancer therapies [6]. Plasma cfDNA analysis is appealing due to the ease with which plasma can be collected and analyzed without prior need of enrichment or isolation of a rare cell population [7]. Beyond mutation analysis, DNA methylation prognostic biomarkers still need to be assessed in liquid biopsy analyses [39]. DNA methylation is considered as an early event in carcinogenesis; thus the detection of tumor‐specific epigenetic alterations in plasma can be used as a screening and/or diagnostic tool in a noninvasive and cost‐effective way. However, large‐scale validation of DNA methylation biomarkers is necessary to boost their implementation into clinical practice [40]. A recent successful bench‐to‐bedside application of DNA methylation analysis is the first blood test based on *SEPT9* promoter methylation that is an FDA‐approved assay for screening and diagnosis of colorectal cancer [41].

It has been recently shown that *KMT2C* is downregulated in NSCLC compared with normal tissues (TCGA data) and that this transcriptional downregulation may be due to its promoter methylation [30]. Based on these very recent findings, we evaluated the clinical relevance of *KMT2C* promoter methylation, as a circulating epigenetic biomarker in plasma cfDNA from NSCLC patients.

Recent data have shown that *KMT2C* is highly mutated on macrodissected lung carcinoids [42]. It has also been recently reported that *KMT2C* is frequently mutated in certain populations with diffuse‐type gastric adenocarcinoma and that *KMT2C* loss promotes epithelial‐to‐mesenchymal transition (EMT) and is associated with worse OS [43]. According to this study, *KMT2C* knockdown promoted EMT and was accompanied by increased expression of N‐cadherin and Slug. This increased migration and invasion in *in vitro* by 47‐ to 88‐fold [43]. These results strongly support our findings, since *KMT2C* loss could result not only through mutations, but DNA methylation as well. Our results, showing a prognostic significance of *KMT2C* promoter methylation in NSCLC, could be possibly explained by the promotion of EMT in these cases.

Epigenetic modifiers are frequently inactivated due to loss‐of‐function mutations and are promising drug targets for lung cancer treatment. In mouse models, lung‐specific loss of Kmt2d promotes lung tumorigenesis and upregulates pathways such as glycolysis [44]. To this end, subsequent pharmacological inhibition of glycolysis suppressed tumor growth *in vitro* and *in vivo* [44]. The detection of low *KMT2C* activity, due to promoter methylation, could specifically have important therapeutic implications. Epigenetic state is altered in bladder cancer cells with low *KMT2C* activity, followed by the decreased expression of DNA damage/repair genes [30]. Therefore, cancer cells with low *KMT2C* activity may be effectively treated with PARP inhibitors that have synthetic lethal effects [45]. Moreover, MLL3 encoded by *KMT2C* enhances the transcription of PD‐L1 and regulates antitumor immunity in mouse xenografts. According to this study, MLL3 interacts with an important immune checkpoint and gives prominence to a hidden therapeutic target [46].

Our findings clearly indicate that the detection of aberrant methylation of the *KMT2C* promoter in plasma‐derived cfDNA is related to reduced DFS and OS in operable NSCLC and reduced PFS and OS in advanced NSCLC. We report a lack of concordance of *KMT2C* promoter methylation in paired primary tumors, their adjacent noncancerous tissues, and corresponding plasma samples at an early stage of NSCLC. In the same group of patients, we observed a higher frequency for *KMT2C* methylation in plasma‐derived cfDNA compared with the corresponding tumor tissues. This could possibly be explained in the context of tumor tissue heterogeneity that is a limitation factor for a representative genomic analysis in primary tissues; tumor cells within the same tumor show distinct phenotypes attributed to genetic and epigenetic alterations. On the other hand, analysis in plasma can reflect tumor heterogeneity, since DNA from different sources is shed in plasma, thus recapitulating tissue's molecular characteristics. In our advanced NSCLC cohort, the Kaplan–Meier analysis has shown a strong correlation between *KMT2C* promoter methylation in plasma cfDNA and poor PFS and OS, which was also verified by the univariate Cox regression analysis.

## 5 Conclusions

To our knowledge, this is the first time that *KMT2C* methylation status is evaluated in NSCLC. Based on the aforementioned, the detection of *KMT2C* promoter methylation in plasma cfDNA provides important prognostic information for NSCLC patients at both early and advanced stages, whereas it is not detected in plasma of healthy individuals. We strongly believe that *KMT2C* promoter methylation in plasma cfDNA merits to be further evaluated and validated as a noninvasive circulating tumor biomarker in a large and well‐defined patient cohort.

## Conflict of interest

The authors declare no conflict of interest.

## Author contributions

SM, AK, and EL conceived and designed the project; ET and VG provided the clinical samples for analysis; SM and IB acquired the data; SM and EL analyzed and interpreted the data; and SM, AK, VG, and EL wrote and edited the original and revised manuscripts.

### Peer Review

The peer review history for this article is available at https://publons.com/publon/10.1002/1878‐0261.12848.

## Supporting information

**Fig. S1.** Kaplan–Meier estimates of unmethylated *KMT2C* operable NSCLC group according to stage (*N* = 28 patients, stage I–II and *N* = 6 patients, stage III–IV). (A) Disease stage in relation to DFS (months) and (B) Disease stage in relation to OS (months).
