# Supplementary figures and images for: KMT2C promoter methylation in plasma‐circulating tumor DNA is a prognostic biomarker in non‐small cell lung cancer
Source: Mol Oncol. 2020 Dec 25;15(9):2412–22. doi: 10.1002/1878-0261.12848 (PMC8410531; doi:10.1002/1878-0261.12848)

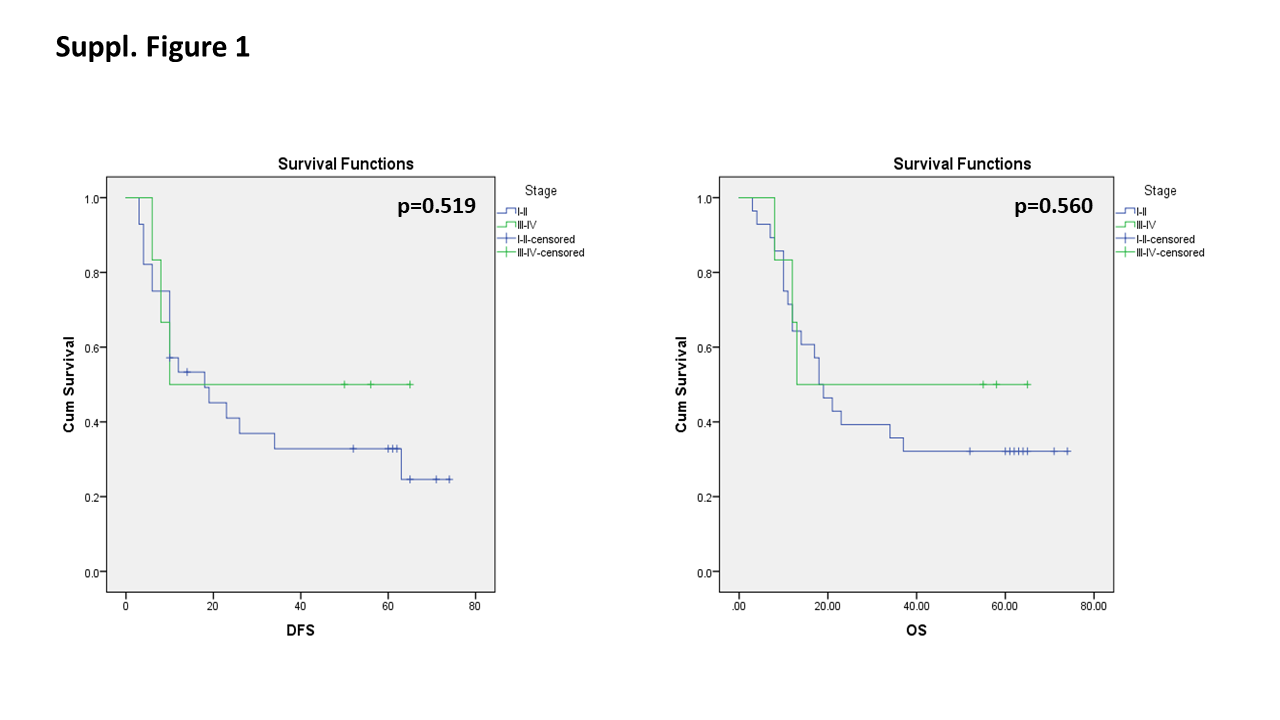

Supplement: Supplementary file 1 — Fig. S1. Kaplan–Meier estimates of unmethylated KMT2C operable NSCLC group according to stage (N = 28 patients, stage I–II and N = 6 patients, stage III–IV). (A) Disease stage in relation to DFS (months) and (B) Disease stage in relation to OS (months). [file MOL2-15-2412-s001.tif]
